# Integrative role of orexigenic peptides in neuroprotection and neurodegenerative disease modulation

**DOI:** 10.1042/BSR20250362

**Published:** 2026-06-29

**Authors:** Zuzana Sopko, Andrea Pačesová, Blanka Železná, Lenka Maletínská

**Affiliations:** Institute of Organic Chemistry and Biochemistry of the Czech Academy of Sciences, 16610 Prague, Czech Republic

**Keywords:** Alzheimer´s disease, neurodegeneration, orexigenic peptides, preclinical models

## Abstract

The neuroprotective properties of several anorexigenic peptides, including leptin and glucagon-like peptide-1, are well established across models of neurodegenerative diseases. However, less is known about the role of orexigenic neuropeptides—including neuropeptide Y, agouti-related peptide, melanin-concentrating hormone, orexins, galanin, and peripherally released hormone ghrelin—that are best known for their role in energy balance and stimulation of food intake. Growing evidence highlights their broader neuroprotective properties across preclinical models of Alzheimer’s disease (AD) and Parkinson’s disease (PD). In AD, these peptides reduce hallmark pathologies such as amyloid burden, tau phosphorylation, oxidative stress, and neuroinflammation, while enhancing synaptogenesis, neurogenesis, and cognitive function. In PD models, ghrelin protects nigrostriatal dopaminergic neurons by restoring autophagic flux, suppressing endoplasmic reticulum stress-mediated apoptosis, and reducing microglial activation, whereas orexin A and B preserve tyrosine hydroxylase expression, promote neuronal excitability, and improve motor and cognitive outcomes. Taken together, these findings position orexigenic peptides as promising modulators of neurodegeneration and highlight their potential as therapeutic targets in AD and PD.

## Introduction

### Orexigenic and anorexigenic peptides: physiological functions and their impact on neurodegeneration

**Figure 1:**
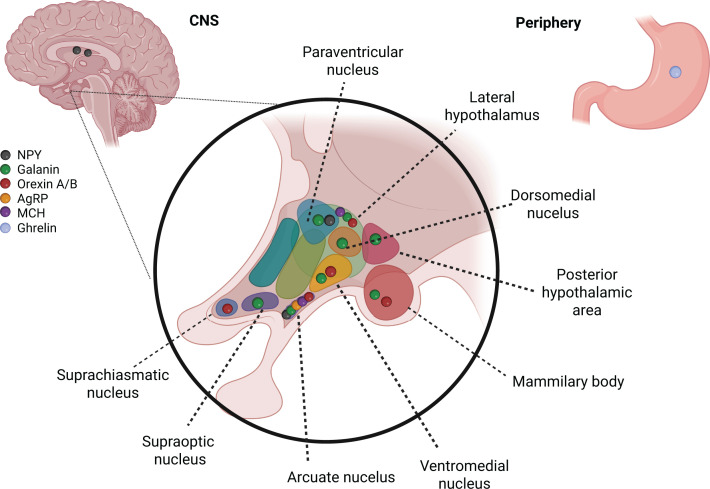
Schematic representation of orexigenic peptide production sites in the CNS and periphery Schematic representation of orexigenic peptide production sites in the CNS and periphery. The illustration highlights hypothalamic nuclei that synthesize NPY, AgRP, MCH, orexins, and GAL, all of which play critical roles in the regulation of appetite, energy balance, and metabolism. Additionally, the stomach is depicted as the source of ghrelin, the only known circulating orexigenic peptide. Together, these central and peripheral signals coordinate food intake, glucose and lipid metabolism, and overall energy homeostasis [5–8].

Orexigenic neuropeptides—including neuropeptide Y (NPY), agouti-related peptide (AgRP), melanin-concentrating hormone (MCH), orexins, and galanin (GAL)—along with the peripherally released orexigenic peptide hormone ghrelin, are primarily known for their role in stimulating food intake and regulating energy homeostasis. Although these peptides typically act in opposite ways to anorexigenic (food intake lowering) peptides in the control of appetite, growing evidence suggests that both groups exert similar neuroprotective effects in models of neurodegenerative diseases such as Alzheimer's disease (AD) [1–4]. More detailed descriptions of anorexigenic neuropeptides as anti-obesity and neuroprotective agents are available in our recent review by Strnadova et al. [1]. Orexigenic neuropeptides are produced in several sites across the central nervous system (CNS) and the periphery, with the hypothalamus serving as a major source; Figure 1 highlights these hypothalamic nuclei alongside the stomach as well as the peripheral origin of ghrelin, released mainly in the stomach, illustrating their coordinated roles in appetite regulation and energy homeostasis.

In addition to their metabolic functions, orexigenic peptides influence CNS pathways involved in memory, plasticity, and neuronal survival. Spatial memory decline and reduced neuron survival contribute to age-related cognitive impairment in AD, with current treatments offering limited benefits. Behavioral studies *in vivo* have demonstrated that these peptides enhance memory formation, possibly by modulating neurotransmitter systems and supporting synaptic plasticity [9–11]. All three studies employed adult rat models with intracerebroventricular (i.c.v.) peptide administration, enabling direct delivery of neuropeptides such as MCH, NPY, and GAL into the brain to assess their central effects on memory, cognition, and neuroprotection [9–11]. Furthermore, they promote neurogenesis and synaptogenesis, processes crucial for maintaining cognitive function and adapting to age-related neuronal loss [11].

AD, the most common cause of dementia, is a progressive neurodegenerative disorder that begins with mild cognitive decline and advances to severe deficits in communication, memory, and daily functioning. Pathologically, AD predominantly affects brain regions involved in cognition, memory, and language processing. It is characterized by the accumulation of amyloid-β (Aβ) plaques and neurofibrillary tangles, accompanied by neuroinflammation, synaptic dysfunction, mitochondrial disturbances, and vascular abnormalities, ultimately leading to neuronal death [12,13].

Parkinson’s disease (PD) is a progressive neurodegenerative disorder primarily characterized by the loss of dopaminergic neurons in the substantia nigra pars compacta, leading to dopamine deficiency in the striatum. A key pathological hallmark is the accumulation of misfolded α-synuclein, forming Lewy bodies that disrupt neuronal function. Mitochondrial dysfunction, oxidative stress, impaired protein degradation, and neuroinflammation further contribute to neuronal damage. These processes collectively result in motor symptoms such as tremor, rigidity, and bradykinesia, as well as non-motor features including cognitive decline and autonomic dysfunction [14,15].

Huntington’s disease (HD) is a hereditary neurodegenerative disorder primarily affecting the striatum, which includes the caudate nucleus and putamen, leading to progressive motor, cognitive, and psychiatric impairments. Affected individuals develop involuntary choreic movements, abnormal postures, and behavioral and cognitive disturbances. The disorder results from a mutation in the huntingtin HTT gene, involving an abnormal expansion of CAG nucleotide repeats that produce a toxic, elongated huntingtin protein. The mutant protein accumulates in neurons, particularly within the striatum, causing cellular dysfunction, neuronal death, and the characteristic symptoms of HD [16,17].

Clinically, this is particularly relevant given the observed metabolic changes in patients with AD. While mid-life obesity is a recognized risk factor for the development of AD, patients often exhibit unintended weight loss [18] and even cachexia by the time dementia manifests [18]. This paradox raises the question of whether orexigenic peptide analogs could serve a dual purpose—addressing both metabolic deficits and cognitive decline in neurodegenerative disorders [2]. Conversely, individuals with obesity, who are at higher risk of AD and other neurodegenerative diseases, might benefit from treatment with anorexigenic peptides, which have also shown neuroprotective properties [3,4,19]. Such an approach underscores the potential of personalized medicine, in which therapeutic strategies are tailored according to a patient’s metabolic and clinical profile. By aligning treatment types—orexigenic or anorexigenic peptide analogs—with individual metabolic states, it may be possible to simultaneously target energy imbalance and neurodegeneration, offering a more comprehensive and patient-specific therapeutic framework for disorders such as AD and PD.

Importantly, emerging human data support the involvement of orexigenic peptide systems in AD. Reduced levels of ghrelin and its active form have been reported in patients with mild cognitive impairment and AD, correlating with cognitive decline [20,21]. Similarly, increased cerebrospinal fluid levels of orexin have been observed in AD patients [22,23], while postmortem studies revealed a reduction in orexinergic neurons in the hypothalamus [24]. In addition, decreased NPY receptor density has been reported in the cortex and hippocampus of AD brains [25], indicating region-specific impairment of orexigenic signaling. Together, these findings suggest that dysregulation of orexigenic peptides is not limited to experimental models but is also present in human AD pathology.

Further research is warranted to clarify the mechanisms of orexigenic neuropeptides and to translate these findings into clinical applications. Nevertheless, by enhancing cognitive processes, stimulating neuronal regeneration, and reducing pathological markers of neurodegeneration, these peptides represent a promising therapeutic avenue for slowing or preventing the progression of disorders such as AD. The present review summarizes the prospective advantages of orexigenic peptides.

### Neuropeptide Y

Neuropeptide Y (NPY) is a 36-amino-acid peptide widely expressed in the mammalian brain, particularly in the striatum and dentate gyrus [7]. It is highly conserved across species and one of the strongest hypothalamic stimulators of appetite [26] under negative energy balance. NPY receptors in the human central and peripheral nervous systems are encoded by distinct genes with diverse tissue distribution and signaling pathways, enabling them to regulate multiple physiological functions primarily through five G protein-coupled receptors (Y1, Y2, Y3, Y5, and Y6). Acting as a neurotransmitter [27], NPY increases food intake and regulates digestion, metabolism, and stress resilience, with receptors distributed in the nervous and gastrointestinal systems [28]. In neurodegenerative diseases such as AD, PD, and HD, changes in NPY levels may represent a compensatory response aimed at providing neuroprotection [29,30]. In the brains of patients with AD, a significant reduction in NPY receptor density has been observed, with decreases of approximately 43% in the temporal cortex and 49% in the hippocampus, suggesting that loss of NPY signaling may be linked to region-specific neurodegenerative processes [25]. Evidence suggests that NPY exerts anti-apoptotic, anti-inflammatory, and pro-phagocytic effects [31], which could help to slow disease progression [30].

New neurons are actively generated throughout life, primarily in the subventricular zone and subgranular zone of the hippocampus and, to a lesser extent, in the hypothalamus, neocortex, striatum, amygdala, and substantia nigra. This ongoing neurogenesis offers potential for restoring neuronal loss in neurodegenerative diseases and is regulated by various factors, including growth factors, cytokines, hormones, and neuropeptides such as NPY. Impaired neurogenesis in the hippocampus is associated with several neurodegenerative diseases, making its enhancement a promising therapeutic strategy. Recent studies by Mirchandani-Duque et al. highlight a functional interaction between NPY and GAL in the limbic system, particularly in regulating neurogenesis [9]. The combined effects of a Y1 receptor (Y1R) agonist and GAL on neurogenesis in the dorsal hippocampus were investigated to explore potential cognitive benefits in Sprague-Dawley rats. Co-administration of GAL and Y1R agonists increased cell proliferation in the dentate gyrus of adult rats, enhanced expression of neuroprotective brain-derived neurotrophic factor (BDNF) and anti-apoptotic factors (B-cell lymphoma 2, Bcl-2) and supported neuron survival and neurite outgrowth in hippocampal neurons. These cellular effects were mediated by Y1R–GALR2 receptor interactions and translated into improved spatial memory in the object-in-place task. The findings support the development of dual-target (Y1R–GALR2) agonists as a novel therapeutic approach for cognitive decline in neurodegenerative diseases [9]. In another study, it was found that NPY administration improved passive avoidance and cognitive memory in Wistar rats treated with i.c.v. of Aβ1–42 (longer form of Aβ peptide, 42 amino acids long) [10]. Recent findings by Ferreira et al indicate direct interactions between NPY and microglia, the innate immune cells of the CNS. Acting primarily through Y1R, NPY inhibits microglial activation, reduces IL-1β release and nitric oxide production, and impairs phagocytosis and motility, underscoring its key role in regulating microglial function during neuroinflammation [32,33].

Moreover, a single i.c.v. administration of NPY prevented depressive-like behavior and spatial memory deficits in Swiss mice subjected to AD-related damage induced by i.c.v. injection of Aβ1–40. These effects were linked to Y2 receptor (Y2R) activation and reduced oxidative stress, without affecting anxiety or locomotor activity [34]. Pharmacological interventions and effects of orexigenic peptides in *in vitro* and *in vivo* models of AD-like/PD-like pathology are stated in Table 1.

**Table 1:** Pharmacological interventions and effects of orexigenic peptides in *in vitro* and *in vivo* models of AD-like/PD-like pathology

Model; age; model establishment	Intervention	Intervention effects	Reference
**Neuropeptide Y**			
• ♂ Swiss mice • 12 weeks • I.c.v. infusion of human Aβ1–40 (shorter form, 40 amino acids long) (400 pmol/mouse)	• NPY: single i.c.v. administration of 0.0234 μmol/μl, 15 min prior to Aβ1–40 administration • BIIE0246: pretreatment with selective Y2 receptor antagonist	• NPY prevented Aβ1–40-induced depressive-like responses • NPY prevented Aβ1–40-induced spatial memory impairments, effect was abolished by Y2 receptor antagonist • NPY blunted the ↑ in lipid peroxidation in hippocampus and prefrontal cortex caused by Aβ1–40	[34]
• ♂ Wistar rats • I.c.v injections of Aβ1–42; 2 μg/μl/side	• NPY: 10 ng/μl, 10 μl, i.c.v, administered 30 min before the retrieval phase of Passive Avoidance Test • Y2 antagonist BIIE-0246 : 20 nM, 200 nM, and 2 μM, i.c.v, administered 15 min before NPY injection in targeted groups	• NPY administration improved memory • Novel object recognition improved after NPY administration • BIIE-0246 had no effect on NPY’s memory improvement	[10]
**Galanin**			
• ♂ Sprague-Dawley rats • 6–8 weeks	• GAL-treated group (3 nmol), Y1R agonist-treated group receiving an NPYY1R agonist [Leu^31^,Pro^34^]NPY (3 nmol), GAL + Y1R: group administered with both substances, and GAL+Y1R+M871: group injected with GAL, [Leu^31^,Pro^34^]NPY, and the GALR2 antagonist (M871, 3 nmol)	• ↑Hippocampal cell proliferation with Y1R agonist and GAL • ↑ Expression of neuroprotective and anti-apoptotic factors: with Y1R agonist and GAL • ↑Survival of neurons with Y1R agonist and GAL • ↑Neurite outgrowth with Y1R agonist and GAL Spatial memory Improved with Y1R agonist and GAL co-injection at 24 h	[9]
• ♂ Sprague-Dawley rats • 6–8 weeks	• Intranasal co-administration of GALR2 agonist M1145 and NPY1R agonist, • administered once daily for three days	• Spatial memory improvement by co-administration of GALR2 and NPY1R agonists • Neuronal survival: Co-administration ↑ survival rate of mature neurons • Neurogenesis: ↑in Doublecortin-labelled cells • Co-administration improved spatial memory and increased the survival rate of mature neurons	[39,40]
**MCH**			
• Primary cortical neurons (embryos were dissected from pregnant C57BL/6J ♀ mice) • ♀ APPswe/PSEN1dE9 Mice (16 weeks) • Scopolamine-Treated Mice (scopolamine IP at a dose of 0.5 mg/kg) • Aβ-injected mice C57BL/6J (i.c.v. injection at 400 pmol of concentration of Αβ1–40), 8 weeks • 5×FAD (16–20 weeks)	• APP/PS1 mice: intranasal (i.n.) administration of MCH at a dose of 1 μg three times a week for 3 months • Scopolamine-treated mice: MCH was administered through the nasal cavity 30 min before scopolamine treatment • Aβ-injected mice: different concentrations of MCH (1 or 5 μg/30 μl) were administered IN to mice daily for 7 days • 5×FAD 100 or 200 nM of MCH for electrophysiology experiments	• Improved memory impairment in both scopolamine-induced memory-impaired mice and Alzheimer’s disease mouse models • ↓ Soluble Aβ in the cerebral cortex of APP/PS1 transgenic mice • Inhibited Aβ-induced cytotoxicity *in vitro* • ↑ LTP in the hippocampus of wild-type and 5×FAD AD mouse models	[111]
**Orexins**			
• ♀ C57BL/6J mice • 9–12 weeks • Housed in an SPF-free vivarium • Experimental autoimmune encephalomyelitis was induced by subcutaneous immunization in the flanks with 100 μg of MOG_35–55_ emulsified with complete Freund's adjuvant supplemented with 5 mg/ml of *Mycobacterium tuberculosis* H37Ra • Mice also received i.p. 300 ng of pertussis toxin on days 0 and 2 post-immunization	• 100 μg of OXA administered intraperitoneally (i.p.) or retro-orbitally (RO) for five consecutive days in preventive or curative settings • 300 μg of OXA administered i.p. or RO for 5 consecutive days in preventive or curative settings	• ↓ Infiltration of pathogenic CD4+ T lymphocytes • Diminished chemokine and cytokine expressions in the CNS • ↓ Demyelination • ↓ Astrogliosis • ↓ Microglial activation • Accumulation of immune cells in lymph nodes • ↑ Foxp3 expression at day 15	[57]
• ♂ MPTP-lesioned PD mice • MPTP (30 mg/kg, i.p. injection) • 8–10 weeks	• I.c.v. application of OXB (600 ng/mouse) or i.c.v. TCS OX2 29 (10 μg/mouse) or mice were pretreated with TCS OX2 29 30 mins before receiving orexin-B and MPTP injection	• OXB exerted excitatory effects via OX2R • Blocking OX2R ↓ the firing rate • ↓ Degeneration of dopaminergic neurons • ↑ General spontaneous activity • Alleviated motor coordination	[60]
**AgRP**			
• Human mesenchymal stem cells (MSCs) were isolated from Wharton’s Jelly • HT22, a mouse hippocampal neuronal cell • C57BL6/J mice, 5×FAD mice	*In vitro :* • HT22 co-treated with 0.25 μM of N-benzoyloxycarbonyl (Z)-Leu-Leu-leucinal (MG-132), a potent proteasome inhibitor, and varying doses of AgRP (0–50 ng/ml) for 24 h. • In vivo : • WJ-MSCs, AgRP (1000 ng/kg), or PBS 1× (vehicle control) were injected into the left hippocampus of both 10–13-month-old C57BL6/J and 5×FAD mice; mice were sacrificed 1 week post injection	*In vitro*: • HT22 that received a combined treatment of MG-132 (1 μM) and AgRP showed a dose-dependent ↓ in ubiquitin protein conjugates ubiquitin-conjugated proteins *In vivo*: • The stereotactic delivery of either WJ-MSCs or AgRP into the hippocampi of C57BL6/J and 5×FAD mice induced ↑ of proteasome activity • ↓ The accumulation of ubiquitin-conjugated proteins • The proteasome activity of WJ-MSC- and AgRP-injected groups was ↑ • The concentration of ubiquitin-conjugated proteins of WJ-MSC- and AgRP-injected groups was ↓ further supporting their roles in the up-regulation of proteasome activity	[69]
**Ghrelin**			
• ♂ 5×FAD mice • 12 weeks	• MK-0677, a ghrelin agonist (I.p., 5 mg/kg daily for 3 weeks)	• ↓ Aβ deposition • ↓ Gliosis • ↑ Neuronal nuclear antigen -positive cells • ↑ Synaptophysin optical density • ↓ Neuroinflammation (Iba-1 and GFAP staining) • ↑ Phosphorylated cAMP response element-binding (pCREB) levels	[83]
• Mouse BV2 microglial cells treated with Aβ1−42 (5 μM) for 48 h co-cultured with mouse hippocampal neuronal cells HT-22 or an indirect co-culture method with a conditioned medium (CM) to co-culture BV2 microglial cells and HT-22 cells • ♂ C57 mice • 8 weeks • Controlled environment with Aβ1–42 treatment; assessment with ferroptosis activator Erastin; behavioral tests and tissue staining for evaluation. • Aβ1–42 (4 μg) injected into the bilateral hippocampi of the mice	• 100 nM, 24 h in cells 24 h after Aβ1–42 injection, ghrelin was administered at a dose of 40–80 μg/kg/day for 7 consecutive days	• ↑ Up-regulation of ferroptosis-related proteins • ↓ Reactive oxygen species and malondialdehyde levels • ↑ Reduced glutathione and superoxide dismutase levels • Alleviation of mitochondrial structural damage • ↓ Pro-inflammatory factors • ↑ Anti-inflammatory factors • Improvement in abnormal behavior • ↓ Neuroinflammation • ↓ Aβ deposition • Modulation of microglial polarization: Ghrelin increased M2 polarization	[82]
• ♂ C57BL/6 mice • MPTP (30 mg/kg/day) • PD mouse model	• I.p.of Ghrelin 2 h before the first MPTP injection • Ghrelin (40, 60, 80 μg/kg/day)	• Ghrelin protected dopaminergic neurons • ↓ Alpha-synuclein accumulation and phosphorylation • Enhanced autophagy by ↑ microtubule-associated protein 1 light chain 3B-II/I and Beclin1 • ↓ Sequestosome 1 (p62) levels • Ghrelin suppressed endoplasmic reticulum stress-related apoptosis pathways • Caspase-12 and caspase-3 activation	[90]
• ♂3×Tg-AD mice	• Subcutaneous injection of Dpr^3^-ghrelin (5 mg/kg)	• ↓ Aβ accumulation, • ↓ Tau hyperphosphorylation • ↓ Neuroinflammatory markers • ↓ Microgliosis	[2]
• Primary cultured hippocampal neurons (prepared from hippocampal E18–E19 Wistar rat embryos) • exposed to AβO (0.5 μM)	• 0.1–0.5 μM rat ghrelin	• ↓ AβO-induced superoxide production • Prevention of mitochondrial membrane depolarization • Improvement of neuronal survival • Inhibition of cell death • Blocking AβO-mediated activation of GSK3β	[77]
• ♂ Wistar rats • Aggregated Aβ1–42 (injected bilaterally into the Cornu Ammonis 3 (CA3) area of the hippocampus 10 μg (2 mg/ml))	• I.p. injection of ghrelin 80 μg/kg for 10 consecutive days	• Improved memory • ↓ Pro-apoptotic protein Bax expression • ↓ Necroptotic proteins RIP1K and RIP3K expressions • ↓ Bax/Bcl-2 ratio • ↓ Autophagic marker Beclin-1 expression	[85]

Abbreviations: I.n., intranasal; I.c.v, intracerebroventricular; Aβ, β-amyloid; i.p., intraperitoneally; APP/PS1, mouse model with APPswe/PSEN1dE9 mutations; NPY, neuropeptide Y; GAL, galanin; OXA, orexin A; CA1, *Cornu ammonis* 1; CA3, *Cornu Ammonis* 3, Aβ1–42, β-amyloid—longer form (42 amino acids long); Aβ1–40, β-amyloid—shorter form (40 amino acids long); AβO, β-amyloid oligomer; RO, retro-orbitally; 5×FAD, familial form of AD; GHSR, ghrelin receptor; p62, sequestosome 1; GALR1–3, galanin receptor 1–3; Iba1, ionized calcium binding adaptor molecule 1, Y1R–Y2R, Y1 and Y2 receptor; MCH, melanin-concentrating hormone; Bax, Bcl-2-associated X protein; Bcl-2; B-cell lymphoma 2; GFAP, glial fibrillary acidic protein; CM, conditioned medium.

### Galanin

GAL, a 29-amino acid neuropeptide, is broadly present in the central and peripheral nervous systems as well as the endocrine system [6]. Its highly conserved amino acid sequence across species highlights its biological significance. GAL functions as a neuromodulator at the synapse through three G-protein-coupled receptors—GALR1, GALR2, and GALR3—enabling its diverse effects throughout the body. GAL is a neuropeptide with neuromodulatory properties, involved in many neurophysiological and behavioral processes, with a key role in regulating anxiety and stress [35]. Administration of GAL in the CNS has repeatedly been shown to produce anxiolytic effects [36,37]. GAL also modulates neurotransmission in the peripheral nervous system, particularly in nociception and pain processing [35]. GAL is abundantly expressed in the hypothalamus, where it increases feeding behavior by influencing energy homeostasis and body weight regulation. It increases fat intake and metabolism via neurons in the anterior paraventricular nucleus that are responsive to circulating insulin and glucose [6,8]. Other hypothalamic GAL populations are involved in water balance through vasopressin interactions and growth regulation *via* growth hormone pathways [6,8]. In addition to these physiological roles, GAL participates in stress regulation, nociception, and neuroendocrine control. Importantly, in the central nervous system, GAL signaling has been implicated in both neurodegeneration and neuroprotection [35], linking its metabolic and behavioral functions to potential therapeutic relevance in brain disorders. In particular, GAL plays a critical role in learning and memory processes and may represent a promising therapeutic tool for neurodegenerative diseases [38].

The study examined by Sanchéz-Varo et al. evaluated the effects of intranasal co-delivery of GALR2 (M1145) and Y1R ([Leu31, Pro34] NPY) agonists in adult Sprague-Dawley rats, showing improved spatial memory, enhanced neuronal survival, and increased differentiation of new neurons. Moreover, this effect is blocked by the GALR2 antagonist M871 [9,39,40]. These effects are linked to the formation of GALR2/Y1R heteroreceptor complexes in neuroblasts, highlighting their potential as therapeutic targets for neurodegenerative diseases like AD’s [9,39,40].

In AD, GAL levels and fiber innervation around basal forebrain cholinergic neurons are markedly increased, but whether this is harmful or protective has been uncertain [41]. Primary basal forebrain neurons cultured from Sprague-Dawley rat fetuses were pretreated with GAL and then exposed to the Aβ. GAL and a GALR2 agonist (AR-M1896) significantly protected cholinergic neurons from Aβ-induced apoptosis and also restored phospho-PKC (protein kinase C) and phospho-Akt (protein kinase B) levels and attenuated cleavage of caspases-3 and caspases-9, supporting a neuroprotective role [41].

On the other hand, in the study of Sundström et al., Sprague-Dawley rats received i.c.v. of GAL or a control solution and were tested in the Morris water maze to assess spatial learning and memory [42]. GAL administration induced a significant deficit in the acquisition of the task but did not affect retrieval or general swimming ability, indicating a selective effect on learning rather than motor performance or recall. GAL has also been shown to suppress acetylcholine release from the ventral hippocampus, suggesting that its impact on learning may be mediated by reduced cholinergic transmission in memory-related circuits. Given the dense cholinergic innervation of the hippocampus and its critical role in spatial memory, these findings supported the idea that GAL modulates cognitive processes by altering hippocampal cholinergic neurotransmission [42].

Eliot-Hunt et al. showed in *in vitro* models, where organotypic hippocampal slices from 5–6-day-old pups and primary hippocampal neurons from 2–3-day-old pups were exposed to glutamate or staurosporine, that GAL-knockout cultures exhibited significantly greater neuronal death, whereas GAL overexpression or treatment with GAL/GALR2 agonist markedly reduced cell loss [43]. *In vivo*, kainate was injected into GAL-knockout and GAL-overexpressing mice to induce excitotoxic hippocampal injury. Knockout mice exhibited markedly increased cell death in CA1 and *Cornu Ammonis 3* (CA3) regions, while GAL-overexpressing mice showed reduced neuronal damage. Together, these results demonstrate that GAL functions as an endogenous neuroprotective factor in the hippocampus and suggest therapeutic potential for GAL-based agonists in brain injury [43]. Pharmacological interventions and effects of orexigenic peptides in *in vitro* and *in vivo* models of AD-like/PD-like pathology are stated in Table 1.

### Melanin-concentrating hormone

MCH is a conserved 19-amino-acid neuropeptide primarily produced in the lateral hypothalamus and zona incerta. Through widespread projections to memory- and emotion-related brain regions—such as the hippocampus, cortex, amygdala, and nucleus accumbens—MCH influences functions including learning, memory, mood, appetite, sleep, and stress regulation. MCH acts via two G protein-coupled receptors, MCHR1 and MCHR2 [44]. MCH promotes lipid accumulation in the liver via the MCHR1 from the lateral hypothalamic area and the parasympathetic nervous system, while it increases fat storage in the adipose through activation of MCHR1 in the arcuate nucleus (ARC) and by suppressing the sympathetic traffic [45]. Activation of MCH-producing neurons has been shown to enhance memory performance and synaptic plasticity, particularly in the hippocampus [11].

Experimental evidence indicates that MCH modulates long-term potentiation (LTP) through N-methyl-d-aspartate (NMDA) receptor-dependent pathways and boosts nitric oxide and cyclic guanosine monophosphate (cGMP) levels, supporting cognitive function [46,47]. Mice carrying the familial AD-linked mutations in amyloid precursor protein (APP) gene (*App*^NL-G-F^ knock-in mice express Swedish (p.LysMet670/671AsnLeu), Beyreuther/Iberian (p.Ile716Phe), and Arctic (p.Glu693Gly) under the endogenous promoter on the C57BL/6J background) showed reduced levels of MCH and abnormal hyperactivity [48,49] in hippocampal neurons characterized by a decrease in neuronal activity of layer 2/3 cortical neurons and an unexpected increase in the frequency of spontaneous Ca^2+^ transients [48,49]. In *App*^NL-G-F^ mice, excessive neuronal activity in the *Cornu Ammonis area 1* (CA1) (reflected by increased spontaneous excitatory postsynaptic current frequency—meaning neurons receive more excitatory inputs at rest) emerges around 2 months, is temporarily normalized by homeostatic mechanisms at ∼4 months, but rises again by ∼6 months as this compensatory process fails as pathology progresses [49]. MCH, produced by sleep-active hypothalamic neurons (a distinct population of neurons in the lateral hypothalamus that are most active during sleep (especially during rapid eye movement (REM) sleep), normally stabilizes hippocampal activity and supports memory by reducing synaptic transmission and neuronal firing in the CA1 [50]. In both APP^NL-G-F^ mice and AD patients, MCH neuron activity and axonal integrity progressively decline, alongside disrupted REM sleep. These findings suggest that MCH system dysfunction contributes to early AD pathology, linking sleep disturbances with impaired synaptic homeostasis and memory [49]. APP/PS1 (mouse model with APPswe/PSEN1dE9 mutations) mice are double transgenic animals expressing a mutated APP with the Swedish mutation (K595N/M596L) and a mutant presenilin-1 (PS1) with the ΔE9 exon deletion, both targeted to neurons in the CNS, with each mutation linked to early-onset AD. In contrast, 5×FAD mice possess a more complex genetic profile, overexpressing human APP 695 with three familial AD mutations—Swedish (K670N/M671L), Florida (I716V), and London (V717I)—alongside human PS-1 carrying two familial AD mutations, M146L and L286V. This combination results in rapid and aggressive Aβ deposition, making 5×FAD mice a robust model for studying AD pathology. MCH was intranasally administered to scopolamine-induced memory-impaired mice and AD (APP/PS1 and 5×FAD) models [51]. MCH improved memory performance, reduced soluble Aβ levels in the cortex of APP/PS1 mice, and enhanced LTP in the hippocampus of both wild-type and AD models. These effects were linked to increased levels of synaptic and neuroplasticity markers such as phosphorylated cyclic adenosine monophosphate response element-binding protein (CREB), glycogen synthase kinase 3 beta (GSK3β), BDNF, tropomyosin receptor kinase B, and postsynaptic density protein 95 that highlight MCH’s potential as a therapeutic agent for treating memory deficits in AD [51]. Pharmacological interventions and effects of orexigenic peptides in *in vitro* and *in vivo* models of AD-like/PD-like pathology are stated in Table 1.

### Orexins

Orexins, comprising orexin A (OXA) and orexin B (OXB), are neuropeptides produced in the lateral hypothalamus and act through two G protein-coupled receptors, OX2R (orexin receptor 1) and OX2R (orexin receptor 2). OXA and OXB are neuropeptides consisting of 33 and 28 amino acids, respectively, sharing 46% sequence identity and derived from the same precursor gene [52]. They regulate various physiological processes, including sleep–wake cycles, energy metabolism (regulating both feeding behavior and energy expenditure) [53], pain modulation, reward, and autonomic functions by projecting widely throughout the CNS, and their activity is strongly influenced by metabolic signals such as leptin, glucose, and ghrelin [8]. Central administration of orexins stimulates feeding, arousal, locomotor activity, and sympathetic drive, particularly during nutritional deficits. In addition, orexin expression in peripheral tissues such as the gastrointestinal tract and pancreas suggests a role in energy homeostasis and insulin regulation [8].

Beyond these roles, the role of orexins in AD remains controversial. While some *in vivo* studies suggest that orexin promotes Aβ accumulation and may exacerbate AD pathology [54], others report neuroprotective effects [55,56], including improved memory, reduced neuroinflammation [56,57], and enhanced synaptic plasticity. This duality highlights a complex, context-dependent function of the orexinergic system in neurodegeneration, warranting further investigation.

In a randomized controlled trial of cognitively unimpaired human participants, the dual orexin receptor antagonist Suvorexant reduced cerebrospinal fluid Aβ levels by ∼10%–20% and decreased tau phosphorylation at threonine-181 by ∼10%–15% compared with placebo. This suggests that Suvorexant, currently approved for insomnia, may have potential for AD prevention, warranting further studies with chronic treatment [58].

In addition, previous studies have shown that OXA exerts neuroprotective effects in PD models. Liu et al. demonstrated that OXA reduced dopaminergic neuron loss and preserved tyrosine hydroxylase (the rate-limiting enzyme in dopamine synthesis, converting the amino acid tyrosine into L-DOPA, which is then transformed into dopamine) expression in the substantia nigra of affected mice. The treatment also improved motor function and spatial memory, accompanied by increased levels of BDNF, a key mediator of neuronal survival and regeneration [59]. In a 1-methyl-4-phenyl-1,2,3,6-tetrahydropyridine (MPTP)-induced mouse model of PD, OXB was found to enhance the firing of nigral dopaminergic neurons through activation of the OX2R, whereas blocking this receptor significantly reduced neuronal activity. Intracerebroventricular administration of OXB not only protected dopaminergic neurons from degeneration but also increased spontaneous activity and improved motor coordination in the parkinsonian mice, suggesting that the neuroprotective effects of OXB in PD may be partly mediated by its excitatory influence on nigral dopaminergic neurons [60]. Moreover, in a similar MPTP-induced mouse model of PD, bilateral microinjection of OXA or OXB increased the spontaneous firing rate of globus pallidus neurons via Ca^2+^ influx through L-type calcium channels, improving motor deficits. OXA primarily acted through OX1R, while OXB acted through OX2R, suggesting receptor-specific pathways in modulating pallidal activity and motor function [61]. Pharmacological interventions and effects of orexigenic peptides in *in vitro* and *in vivo* models of AD-like/PD-like pathology are stated in Table 1.

On the other hand, extensive *in vivo* studies have implicated the orexinergic system in the pathogenesis of AD, particularly regarding Aβ production and accumulation [54]. The study used both wild-type C57BL/6 mice and human APP transgenic mice (Tg2576) to examine how sleep–wake cycles and orexin signaling influence Aβ metabolism and deposition. Using *in vivo* hippocampal microdialysis, interstitial fluid (ISF) Aβ levels were continuously measured under varying sleep conditions. They observed that ISF Aβ levels increased during wakefulness and sleep deprivation and decreased during sleep or after administration of the dual orexin receptor antagonist (DORA) almorexant. Conversely, i.c.v. injection of OXA elevated ISF Aβ levels by promoting wakefulness. Chronic sleep restriction for 21 days significantly increased Aβ plaque deposition in Tg2576 mice, whereas systemic DORA treatment for 8 weeks reduced plaque burden across cortical and hippocampal regions. These findings demonstrated that orexin signaling and sleep–wake regulation directly modulate Aβ accumulation, suggesting that enhancing sleep through orexin receptor blockade could mitigate AD pathology [54].

OXA suppressed autophagy in an intracerebral hemorrhage rat model *via* the OXR1-mediated extracellular signal-regulated kinase/mammalian target of rapamycin (ERK/mTOR) signaling pathway to exert neuroprotective effects, and it might provide a novel therapeutic approach in patients suffering from intracerebral hemorrhage [55]. Loss of orexin signaling is linked to cognitive impairment, neuroinflammation, and neurodegeneration. In orexin-deficient (O/A3) mice, memory performance was significantly impaired and associated with increased hippocampal microglial activation, highlighting a neuroprotective role for orexin in maintaining cognitive function [62]. OXB alleviates chronic constriction injury-induced neuropathic pain by reducing microglial activation, inflammation, and oxidative stress in the spinal cord. These effects were mediated through down-regulation of proinflammatory markers and inhibition of Jun N-terminal kinase/nuclear factor kappa-light-chain-enhancer of activated B cells signaling, suggesting a neuroprotective role for OXB [63].

### Agouti-related peptide

AgRP is a 132-amino acid neuropeptide produced by NPY/AgRP neurons in the ARC, where it co-localizes with NPY and projects to multiple hypothalamic regions involved in energy homeostasis [8]. AgRP functions as a potent endogenous antagonist of melanocortin-3 and melanocortin-4 receptors, key regulators of energy balance, and its activation promotes hyperphagia, reduced thermogenesis, and increased energy efficiency [64,65]. In a study by Fan et al., male C57BL/6J mice—including wild-type, leptin-deficient (*ob/ob*), and yellow agouti (*A^y^*) strains—were used to investigate melanocortin receptor signaling in the regulation of feeding. Intracerebroventricular injections of the melanocortin agonist MTII potently inhibited feeding in multiple models of hyperphagia, whereas co-administration of the agouti-mimetic antagonist SHU9119 or agouti peptide blocked this effect. Conversely, SHU9119 alone significantly enhanced nocturnal and fasting-induced food intake, demonstrating that tonic activation of melanocortin-3 and -4 receptors suppresses feeding behavior. Chronic disruption of this pathway, as seen in *A^y^* mice, reproduced the obesity phenotype characterized by hyperphagia, hyperinsulinemia, and late-onset obesity. These results established that antagonism of hypothalamic melanocortin signaling—mediated by AgRP or agouti peptides—drives sustained increases in food intake and adiposity [65]. AgRP expression is tightly regulated by metabolic status: leptin suppresses expression of AgRP, while fasting and leptin deficiency up-regulate it [66]. Altered AgRP levels have been observed in obesity and diet-induced metabolic changes, underscoring its critical role in energy homeostasis [67,68].

Notably, the ubiquitin proteasome system, essential for protein degradation, is impaired in neurodegenerative diseases like AD, contributing to toxic protein accumulation. A study by Lee et al. explored the potential of the recombinant human AgRP, secreted by human Wharton’s Jelly-derived mesenchymal stem cells (WJ-MSCs), the gelatinous connective tissue that encases the two arteries and single vein within the umbilical cord, to enhance proteasome activity in AD [69]. *In vivo*, delivery of WJ-MSCs or AgRP into the hippocampus of wild-type and 5×FAD mice enhanced proteasome activity and reduced ubiquitin-conjugated protein accumulation; thus, WJ-MSCs and AgRP can modulate proteasome function, highlighting a promising therapeutic strategy to reduce protein aggregation and potentially slow AD progression [69]. Researchers developed a mouse model in which inhibitory neurons expressing AgRP can be selectively destroyed by administering diphtheria toxin (DT) to animals engineered to express the human DT receptor in the AgRP gene locus. Loss of AgRP neurons caused starvation and triggered strong activation of the Fos gene in brain regions they normally innervate, such as the ARC, paraventricular nucleus, medial preoptic area, lateral septum, and nucleus of the solitary tract. Along with these changes, the robust increase in glial fibrillary acidic protein staining (astrocytes) was observed as well as ionized calcium binding adaptor molecule 1 and cluster of differentiation 11b staining (microglia) in the ARC in response to AgRP neuron ablation, likely due to excitotoxicity from loss of inhibitory input [70]. Pharmacological interventions and effects of orexigenic peptides in *in vitro* and *in vivo* models of AD-like/PD-like pathology are stated in Table 1.

### Ghrelin: a multifunctional orexigenic peptide with neuroprotective potential

Ghrelin, the only known peripheral orexigenic hormone, is a 28-amino acid brain–gut hormone predominantly produced by the gastrointestinal tract that can cross the blood–brain barrier [71] and act within the CNS. It binds to the growth hormone secretagogue receptor (GHSR), which is widely expressed in the brain and periphery and shows higher expression during early developmental stages [72]. It is the only known circulating hormone that stimulates appetite, increasing food intake and body weight while opposing leptin’s inhibitory effects. Ghrelin levels rise before meals, fall after eating, and fluctuate with metabolic states such as fasting, obesity, anorexia nervosa, and bulimia. By activating NPY/AgRP neurons in the hypothalamus, it drives feeding behavior and fat storage [73]. Overall, ghrelin serves as a crucial regulator of appetite and long-term energy balance.

While ghrelin is best known for regulating energy balance and neuroendocrine functions, it also plays a crucial role in higher cognitive processes, including learning and memory. Its influence on mitochondrial function, synaptic integrity, and neuroinflammation makes it a promising target for the treatment of neurodegenerative diseases such as PD, stroke, epilepsy, and particularly AD.

Multiple lines of evidence implicate ghrelin in the pathophysiology of AD. A single-nucleotide polymorphism in the ghrelin gene (rs4684677, Leu90Gln), which results in a structural alteration of the peptide and may modulate its neuroprotective and metabolic functions, has been associated with an earlier onset of AD [74]. Furthermore, decreased ghrelin mRNA expression has been detected in the temporal gyrus of individuals with AD [75].

Experimental studies demonstrate that ghrelin or its agonists can reduce Aβ accumulation, protect against methylglyoxal-induced neuronal apoptosis, and improve cognitive performance in AD models [73,76,77]. In the study by Martins et al. [77], primary cultured hippocampal neurons were exposed to synapto- and neurotoxic Aβ oligomers (AβO) to examine whether ghrelin could counteract AβO-induced hippocampal dysfunction. The authors showed that ghrelin, acting through its receptor, reduced AβO-induced superoxide production, prevented mitochondrial membrane depolarization, improved neuronal survival, and inhibited cell death while also blocking AβO-mediated activation of GSK3β [77].

Mechanistically, ghrelin’s neuroprotective actions are multifaceted. It inactivates microglial inflammasomes, suppresses pro-inflammatory cytokine release, reduces oxidative stress, and limits apoptosis [78,79]. Ferroptosis, a form of programmed cell death driven by disrupted iron homeostasis, is thought to play a role in the development of neurological, particularly neurodegenerative, diseases [80,81]. In Aβ1–42-stimulated AD mouse model, ghrelin mitigates neuroinflammation, ferroptosis, and cognitive deficits, partly by promoting a shift in microglial polarization from the pro-inflammatory M1 phenotype to the anti-inflammatory M2 phenotype [82]. These beneficial effects are reversed by ferroptosis activation, highlighting the role of ferroptosis inhibition in ghrelin-mediated neuroprotection. In another study, MK-0677, a potent ghrelin receptor agonist, was tested in 5×FAD transgenic mice to evaluate its effects on Aβ pathology [83]. The treatment reduced Aβ accumulation, gliosis, and neuronal and synaptic loss, while preserving phosphorylated CREB levels in the hippocampus. These results indicate that MK-0677 may have therapeutic potential in early-stage AD by mitigating Aβ burden, neuroinflammation, and neurodegeneration [83]. Moreover, in a triple transgenic mouse model of AD (3×Tg-AD), the stable ghrelin analogue Dpr^3^-ghrelin reduced intraneuronal Aβ deposits in the hippocampus and amygdala. It also decreased microgliosis in the hippocampus, amygdala, and cortex, suggesting an anti-inflammatory effect [2].

In a rat model of Aβ-induced cognitive impairment, intraperitoneal (i.p.) ghrelin administration for ten days improved performance in the Morris water maze and passive avoidance tests. Ghrelin reduced malondialdehyde levels in both hippocampus and serum, prevented Aβ-induced increases in lipid peroxidation, and restored hippocampal catalase activity suggesting ghrelin function in protection against hippocampal oxidative damage and supports antioxidant defences [84]. Malondialdehyde is a reactive by-product of polyunsaturated fatty-acid oxidation and a widely used marker of oxidative stress; elevated levels can damage proteins, DNA, and cell membranes, thereby contributing to neuronal dysfunction and cell death [84]. In the study by Naseri et al. [85], an AD-like model was established in adult male Wistar rats by bilateral intra-hippocampal CA3 injection of aggregated Aβ1–42, which induced hippocampal neurodegeneration and cognitive impairment. Intraperitoneal administration of ghrelin for 10 consecutive days improved memory and reduced pro-apoptotic and necroptotic protein expression while increasing Beclin-1. These findings suggest ghrelin protects neurons by inhibiting cell death and promoting autophagy [85] which is particularly relevant given that recent research has identified autophagy dysfunction in numerous metabolic and degenerative conditions, including metabolic dysfunction-associated steatohepatitis, cardiovascular disease, neurodegenerative disorders, renal and pulmonary diseases, gastrointestinal disorders, and various inflammatory disorders [86].

Importantly, using the 3×Tg-AD mouse model of AD, both anorexigenic (palm^11^-PrRP) and orexigenic peptides (Dpr^3^-ghrelin) have been shown to attenuate the hallmarks of neurodegeneration. These include a reduction in Aβ accumulation, tau hyperphosphorylation, and neuroinflammatory markers, all of which are central to the pathophysiology of AD [2]. This neuroprotective profile indicates that orexigenic peptides may act through common molecular pathways as anorexigenic peptides that mitigate neuronal damage and support cognitive resilience. Together, these studies position ghrelin as a key modulator of AD-related neuropathology, with therapeutic potential through its combined effects on protein aggregation, mitochondrial function, oxidative stress, inflammation, and synaptic maintenance [79].

Although several studies have reported neuroprotective effects of ghrelin in AD models, evidence regarding its impact on Aβ pathology remains inconclusive. In the study by Moon et al., treatment of 3-month-old 5×FAD mice with ghrelin for one month did not significantly alter Aβ plaque load in the hippocampus, as measured by the 4G8 antibody that recognizes amino acids 17-24 of the Aβ peptide, despite a trend toward reduction. While ghrelin increased doublecortin-positive neuroblasts in the dentate gyrus, this enhancement of neurogenesis occurred without measurable changes in Aβ deposition [87]. Similarly, another study using MK-0677, a potent ghrelin receptor agonist, in 5×FAD mice found that although hippocampal neurogenesis was stimulated, there was little to no prevention of hippocampal Aβ accumulation, synaptic loss, microglial activation, or cognitive decline. Moreover, MK-0677 treatment at higher doses significantly increased mortality in 5×FAD mice [88,89]. Together, these findings suggest that while ghrelin signaling can promote neurogenesis, its ability to mitigate Aβ pathology or prevent cognitive deficits in AD models is limited in some studies, underscoring the need for further investigation into its therapeutic potential and mechanisms.

In an MPTP-induced PD mouse model, ghrelin protected dopaminergic neurons, reduced alpha-synuclein accumulation and phosphorylation, and enhanced autophagy by increasing microtubule-associated protein 1 light chain 3B-II/I and Beclin1 (a key molecule involved in autophagic flux initiation and autophagosome formation) while decreasing sequestosome 1 (p62) levels. Ghrelin also suppressed endoplasmic reticulum stress-related apoptosis pathways, including inositol-requiring enzyme 1 alpha, caspase-12, and caspase-3 activation, and thus the findings suggest ghrelin exerts neuroprotection in PD by modulating alpha-synuclein, promoting autophagy, and inhibiting endoplasmic reticulum stress-mediated apoptosis [90]. In 6-hydroxydopamine–induced PD models, ghrelin reduced motor deficits, preserved tyrosine hydroxylase expression, and improved cell survival. It enhanced autophagic flux by increasing autophagy-related protein 7 and microtubule-associated protein 1 light chain 3-II, decreasing p62, restoring lysosome function, and reversing depletion of transcription factor EB, while also lowering apoptosis markers such as Bcl-2-associated X protein and cleaved caspase-3. Blocking autophagy or lysosome function weakened these effects, and transcription factor EB knockdown eliminated ghrelin’s anti-apoptotic action, suggesting that ghrelin protects dopaminergic neurons by restoring transcription factor EB–mediated autophagic flux [91]. Pharmacological interventions and effects of orexigenic peptides in *in vitro* and *in vivo* models of AD-like/PD-like pathology are stated in Table 1.

## Summary and future directions

Orexigenic neuropeptides NPY, AgRP, MCH, orexins, GAL, and peripheral peptide hormone ghrelin have been increasingly recognized as modulators of core pathologies in AD models. They were shown to reduce Aβ burden, attenuate neuroinflammation, and improve synaptic function and memory, suggesting convergent neuroprotective effect.

Regarding Aβ pathology, MCH reduced levels of soluble Aβ in the cortex of APP/PS1 mice [51], AgRP enhanced proteasome activity in 5×FAD mice [69], and Dpr^3^-ghrelin reduced intraneuronal Aβ plaques in the 3×Tg-AD mouse model [2].

Neuroinflammation, another hallmark of AD, involves the transition of microglia from a protective ‘resting’ state that clears Aβ and supports neurons to an activated amoeboid form that drives synaptic loss, tau pathology, and release of pro-inflammatory mediators. NPY was shown to inhibit microglial activation, reduce IL-1β release, and nitric oxide production, underscoring its regulatory role in neuroinflammation [32,33]. Similarly, ghrelin mitigated neuroinflammatory responses in mice after Aβ1–42 stimulation [82], while the stable analogue Dpr^3^-ghrelin reduced microgliosis in hippocampus, amygdala, and cortex of 3×Tg-AD mice [2], pointing to its anti-inflammatory effect.

Impaired neurogenesis in the hippocampus is associated with several neurodegenerative diseases, making the enhancement of new neurons a promising therapeutic strategy. In the study by Moon et al., treatment of 5×FAD mice with ghrelin increased a number of doublecortin-positive neuroblasts in the dentate gyrus, indicating enhanced neurogenesis [87]. Moreover, co-administration of GALR and Y1R agonists increased neuron proliferation in the dentate gyrus of adult rats, enhanced expression of BDNF and anti-apoptotic proteins, and supported neuron survival and neurite outgrowth in hippocampal neurons [9]. Intranasal co-delivery of GALR2 (M1145) and Y1R agonists in adult Sprague-Dawley rats showed enhanced neuronal survival and increased differentiation of new neurons [9,39,40].

Memory impairment develops in AD, PD, and other neurodegenerative diseases. In Aβ1–42-stimulated mice, ghrelin mitigated cognitive deficits, partly by promoting a shift in microglial polarization from the pro-inflammatory M1 phenotype to the anti-inflammatory M2 phenotype [82]. In a chronic unpredictable mild stress mouse model, acyl ghrelin administration alleviated depression-like behaviors and restored hippocampal neurogenesis and spine density through GHSR signaling [92]. In primary cultured hippocampal neural stem cells, acyl ghrelin promoted proliferation via the phosphoinositide 3-kinase pathway, an effect blocked by the GHSR antagonist D-Lys^3^-GHRP-6, indicating that ghrelin maintains hippocampal integrity and resilience to stress through receptor-dependent mechanisms [92]. Co-delivery of GALR2 (M1145) and Y1R agonists in adult Sprague-Dawley rats resulted in improved spatial memory [9,39,40]. NPY administration improved passive avoidance and cognitive memory in Wistar rats stimulated with i.c.v. of Aβ1–42 (longer form, 42 amino acids long) [10]. Moreover, a single i.c.v. dose of NPY prevented depressive-like behavior and spatial memory impairments induced by Aβ1–40 in Swiss mice [34]. MCH was intranasally administered to scopolamine-induced memory-impaired mice and AD (APP/PS1 and 5×FAD) mouse models, where it improved memory performance in both AD models, enhanced LTP in the hippocampus of both wild-type and AD mice, and increased levels of synaptic and neuroplasticity markers in APP/PS1 mice [51]. Ghrelin directly activates NPY/AgRP neurons in the hypothalamus, and its neuroprotective effects in AD models—such as promotion of autophagy [85,93] and suppression of neuroinflammation [2,85]—are likely mediated at least in part through NPY signaling. Notably, NPY and its receptors (NPYR) are abundantly expressed in the hippocampal formation [94], where they modulate cognitive functions, while GHSRs are also highly localized in this region (but not ghrelin itself) [95]. This shared hippocampal distribution supports the possibility of a functional ghrelin–NPY axis contributing to hippocampal neuroprotection and memory regulation. Indeed, NPY has been shown to mediate ghrelin-induced autophagy in cortical neuron cultures [96], supporting a functional similarity between the two peptides. Because autophagy declines with aging and in age-related neurodegenerative diseases, the synergistic action of NPY and ghrelin in promoting autophagy may represent a potential strategy to slow down the aging process. Moreover, Xu et al. reported that ghrelin induces hypothalamic NPY production via the 5' adenosine monophosphate-activated protein kinase–mTOR pathway, which provides a mechanistic basis for ghrelin–NPY interaction [97].

Even though MK-0677 (ibutamoren), a potent oral non-peptidic GHSR agonist, showed promise by stimulating growth hormone release and insulin-like growth factor 1 (IGF-1) production, and preclinical studies suggested possible neuroprotective effects [83], its translation into AD therapy has been disappointing. A large randomized controlled clinical trial found that while MK-0677 increased circulating IGF-1 levels, it did not slow cognitive decline or alter disease progression in AD patients [98]. Similarly, in 5×FAD transgenic mice, MK-0677 enhanced hippocampal neurogenesis but failed to prevent Aβ deposition, synaptic loss, gliosis, or memory deficits [99]. Moreover, higher doses were linked to increased mortality in experimental models.

Another important consideration is delivery and pharmacokinetics. Natural acyl-ghrelin has a very short plasma half-life (∼9–13 min intravenously [100]) due to rapid deacylation and clearance, which limits the effectiveness of intermittent intraperitoneal dosing in experimental models [85]. Although MK-0677 was developed as a long-acting, orally available ghrelin mimetic, its failure to modify AD pathology suggests that receptor stimulation alone is insufficient and that optimized pharmacokinetics must be coupled with broader mechanistic actions. This underlines the need for novel analogs or alternative delivery routes, such as intranasal or controlled-release systems, to achieve sustained receptor engagement in brain circuits relevant to AD and PD. These findings underscore the need to move beyond MK-0677 and ghrelin alone and focus on combinatorial strategies or the development of stable ghrelin analogs, such as in the study by Mengr et al. [2,76] or [101–103], with broader mechanistic actions and improved pharmacological profiles.

Relevance extends to PD, where orexigenic signaling—particularly ghrelin and orexins—provides nigrostriatal protection. In PD models induced by toxins such as MPTP and 6-hydroxydopamine, ghrelin preserves dopaminergic neurons in the substantia nigra pars compacta and striatum by restoring autophagic flux, inhibiting endoplasmic reticulum–stress apoptosis, and reducing microglial activation [90]. Complementary findings show that OXA protects dopaminergic neurons, preserves tyrosine hydroxylase expression, and improves motor and memory function [59], while OXB enhances firing of nigral dopaminergic neurons through OX2R activation, leading to improved motor coordination [60]. Together, these peptides emerge as candidates for modifying striatal vulnerability and motor outcomes in PD. Ghrelin stimulates neurons in the ARC (notably NPY/AgRP neurons), which in turn project to orexin-producing neurons in the lateral hypothalamus, suggesting indirect ghrelin-mediated activation of the orexin system and thus proposing the hypothesis that ghrelin may support orexin function (through upstream activation), and together these systems could protect dopaminergic neurons in PD models by enhancing autophagy, reducing apoptosis and inflammation, preserving tyrosine hydroxylase and neuronal activity, and improving motor function [59,60,90,91].

**Figure 2:**
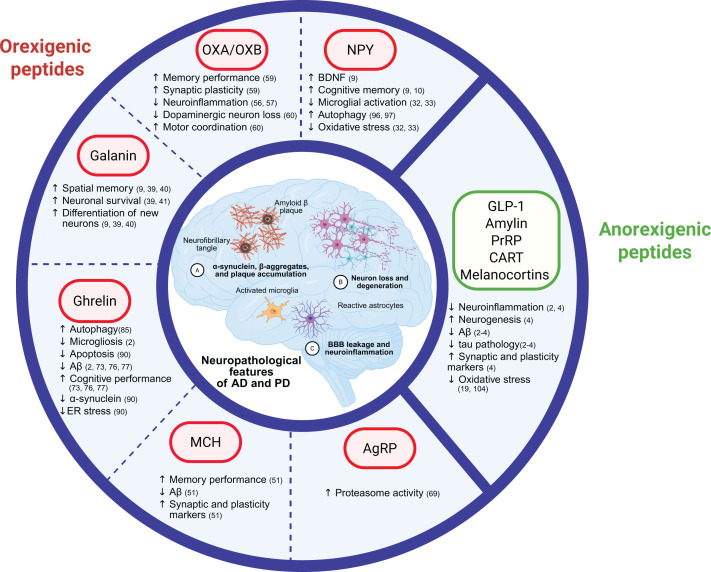
Neuroprotective effects of orexigenic and anorexigenic peptides in Alzheimer’s and Parkinson’s disease-like pathology The scheme illustrates how orexigenic peptides—ghrelin, OXA/OXB, NPY, GAL, MCH, and AgRP—and anorexigenic peptides—GLP-1, amylin, prolactin-releasing peptide (PrRP), cocaine- and amphetamine-regulated transcript (CART), and melanocortins—influence key neuropathological features of AD and PD.

Although orexigenic and anorexigenic peptides act as physiological antagonists in food intake regulation, accumulating evidence suggests convergence in their neuroprotective mechanisms. A shared hallmark could be inflammation control: ghrelin, NPY, glucagon-like peptide-1 (GLP-1) analogues, and others reduce microgliosis and inflammatory markers in transgenic AD models, highlighting immune modulation as a shared therapeutic axis despite their opposite metabolic effects [2–4,19,104]. In PD, particularly ghrelin and orexins provide nigrostriatal protection in the substantia nigra pars compacta and striatum through restored autophagic flux, inhibition of endoplasmic reticulum stress–mediated apoptosis, and reduced microglial activation, while anorexigenic GLP-1 receptor agonists likewise protect nigrostriatal pathways via anti-inflammatory and trophic mechanisms [104–106]. Beyond neuroinflammation, another common feature emerging from both orexigenic and anorexigenic peptide analogues is their ability to promote synaptogenesis and neurogenesis, processes that are impaired in AD and strongly linked to cognitive decline [4,87,104,107]. This overlap suggests that despite opposing metabolic roles, these peptides may converge on shared pathways of neuronal repair and plasticity, highlighting their therapeutic potential in neurodegenerative disorders. Neuroprotective effects of orexigenic and anorexigenic peptides in AD and PD-like pathology are summed up in Figure 2.

Personalized medicine offers a promising direction for neurodegenerative disorders by tailoring therapeutic strategies to the individual metabolic and clinical profile of each patient. In AD, for example, some patients present with cachexia and unintended weight loss, whereas others experience midlife obesity—both conditions that can influence disease progression and treatment response [108,109]. This variability opens the possibility of using orexigenic peptides to support patients in catabolic or cachectic states, while anorexigenic peptide analogs may benefit those with obesity or metabolic dysfunction, as described throughout our recent review [110]. Beyond metabolic correction, these peptides also share neuroprotective properties—such as reducing neuroinflammation, enhancing synaptic plasticity, and supporting neuronal survival—making them particularly suited for individualized treatment strategies. Ultimately, integrating metabolic status with molecular targeting of neuroprotective pathways may represent a crucial step toward truly personalized interventions for AD and related neurodegenerative conditions.

In summary, orexigenic peptides positively influence Aβ dynamics, neuroinflammation, and nigrostriatal resilience, offering mechanistic entry points for both AD and PD. Yet, clinical translation will likely require multimodal strategies.

## Abbreviations

3×Tg-AD: the triple transgenic mouse model of Alzheimer´s disease
5×FAD: familial form of AD
AD: Alzheimer’s disease
AgRP: agouti-related peptide
APP: amyloid precursor protein
APP/PS1: mouse model with APPswe/PSEN1dE9 mutations
ARC: arcuate nucleus
Aβ: β-amyloid
Aβ1–40: β-amyloid—shorter form (40 amino acids long)
Aβ1–42: β-amyloid—longer form (42 amino acids long)
AβO: β-amyloid oligomer
Bcl-2: B-cell lymphoma 2
BDNF: brain-derived neutrotrofic factor
CA1: *Cornu ammonis 1*
CA3: *Cornu Ammonis 3*
cGMP: cyclic guanosine 3',5'-monophosphate
CNS: central nervous system
CREB: cAMP response element-binding
DORA: dual orexin receptor antagonist
Dpr^3^-ghrelin: lipidized ghrelin analogue
DTd: iphtheria toxin
ERK: extracellular signal-regulated kinase
GAL: galanin
GHSR: ghrelin receptor
GLP-1: glucagon-like peptide-1
GSK3β: glycogen synthase kinase 3 beta
HD: Huntington’s disease
i.c.v.: intracerebroventricular
i.n.: intranasal
i.p.: intraperitoneally
IGF-1: insulin-like growth factor 1
ISF: interstitial fluid
LTP: long-term potentiation
MCH: melanin-concentrating hormone
MK-0677: ghrelin receptor agonist
MPTP: 1-methyl-4-phenyl-1,2,3,6-tetrahydropyridine
mTOR: mammalian target of rapamycin
NMDA: N-methyl-d-aspartate
NPY: Neuropeptide Y
O/A3: orexin-deficient mice
OX1R: orexin receptor 1
OX2R: orexin receptor 2
OXA: orexin A
OXB: orexin B
p62: sequestosome 1
PD: Parkinson’s disease
PKC: protein kinase C
PS1: presenilin-1
PSEN1dE9: Mutation in human presenilin 1 carrying the exon-9-deleted variant
REM: rapid eye movement
WJ-MSCs: human Wharton’s Jelly-derived mesenchymal stem cells
Y1R: Y1 receptor
Y2R: Y2 receptor
